# Noninvasive Prenatal Testing: Comparison of Two Mappers and Influence in the Diagnostic Yield

**DOI:** 10.1155/2018/9498140

**Published:** 2018-06-07

**Authors:** Irene Gómez-Manjón, Ana Moreno-Izquierdo, Sonia Mayo, Marta Moreno-García, Aitor Delmiro, David Escribano, F. Javier Fernández-Martínez

**Affiliations:** ^1^Genetics and Inheritance Research Group, Instituto de Investigación Hospital Universitario 12 de Octubre (i+12), Avda. de Córdoba s/n, Madrid 28041, Spain; ^2^Division of Prenatal Diagnosis, Department of Genetics, Hospital Universitario 12 de Octubre, Avda. de Córdoba s/n, Madrid 28041, Spain; ^3^Department of Biochemistry, Hospital Universitario 12 de Octubre, Avda. de Córdoba s/n, Madrid 28041, Spain; ^4^Centro de Investigación Biomédica en Red de Enfermedades Raras (CIBERER), U723 Madrid, Spain; ^5^Fetal Medicine Unit, Department of Obstetrics and Gynaecology, Hospital Universitario 12 de Octubre, Avda. de Córdoba s/n, Madrid 28041, Spain

## Abstract

**Objective:**

The aim of this study was to determine if the use of different mappers for NIPT may vary the results considerably.

**Methods:**

Peripheral blood was collected from 217 pregnant women, 58 pathological (34 pregnancies with trisomy 21, 18 with trisomy 18, and 6 with trisomy 13) and 159 euploid. MPS was performed following a manufacturer's modified protocol of semiconductor sequencing. Obtained reads were mapped with two different software programs: TMAP and HPG-Aligner, comparing the results.

**Results:**

Using TMAP, 57 pathological samples were correctly detected (sensitivity 98.28%, specificity 93.08%): 33 samples as trisomy 21 (sensitivity 97.06%, specificity 99.45%), 16 as trisomy 18 (sensibility 88.89%, specificity 93.97%), and 6 as trisomy 13 (sensibility 100%, specificity 100%). 11 false positives, 1 false negative, and 2 samples incorrectly identified were obtained. Using HPG-Aligner, all the 58 pathological samples were correctly identified (sensibility 100%, specificity 96.86%): 34 as trisomy 21 (sensibility 100%, specificity 98.91%), 18 as trisomy 18 (sensibility 100%, specificity 98.99%), and 6 as trisomy 13 (sensibility 100%, specificity 99.53%). 5 false positives were obtained.

**Conclusion:**

Different mappers use slightly different algorithms, so the use of one mapper or another with the same batch file can provide different results.

## 1. Introduction

The prevalence in Europe of unbalanced chromosome abnormality in the birth rate, between the years 2000 and 2006, was 43.8/10000 births [1]. Diagnostic testing of these diseases currently requires invasive procedures, which means an additional risk of fetal loss that varies between 0.48 and 1.36% [2]. Therefore, medical professionals have tried to improve the methods of screening prior to these invasive tests, developing the noninvasive prenatal testing (NIPT) [3]. This kind of test uses cell-free fetal DNA (cffDNA) in maternal plasma [4]. This DNA is mainly derived from the cytotrophoblasts of chorionic villi in placenta [5]. It is estimated that cffDNA represents 3-10% of circulating cell-free DNA (cfDNA) in maternal plasma and can be detected in the first trimester of pregnancy, increasing in abundance as the placenta grows [6].

Some clinical applications of cffDNA are fetal sex determination, Rhesus D genotyping, autosomal dominant monogenic diseases, and noninvasive prenatal detection of chromosome aneuploidy [7]. To date, there is no consensus on the best way to detect fetal aneuploidy using fetal nucleic acids from the maternal circulation, whole genome sequencing, or target sequencing [8]. In both, millions of short DNA fragments are sequenced simultaneously and aligned against a human genome reference, using different algorithms. Then, the amount of sequences produced from different chromosomes is compared to detect any small overrepresentation caused by a fetal aneuploidy [9].

In the analysis of high-throughput sequencing for NIPT of aneuploidies, the most critical computational process is the alignment (mapping) of the generated reads to the reference sequence. Therefore, a mapper has to be sensitive and accurate and should be able to balance speed and memory usage. The main goal of a mapper is to find the true location of each read on a reference genome and, ideally, distinguish between natural genetic variations and technical sequencing errors. There are many read mappers, which use distinct algorithmic approaches and slightly different definitions of the read mapping. Consequently, the outcome of an analysis may differ significantly depending on the way reads are mapped.

The available alignment solutions are based on different programming approaches, dynamic programming, and backward search techniques. For example, the computational complexity of dynamic programming approaches, such as Smith-Waterman Algorithm or the Hidden Markov Models, depends on the length of the read multiplied by the length of the reference genome. However, the computational complexity of backward search techniques, based on the Burrows-Wheeler Transform, depends only on the length of the read.

The number of next-generation sequencing read mappers has been growing rapidly in recent years, so determining which mapper is the most suitable for a specific application is not simple [10]. In this study two different read mappers are compared to determine which one works better in noninvasive prenatal detection of common aneuploidies using semiconductor sequencing technology: TMAP (Torrent Mapping Alignment Program) developed by Nils Homer et al. from Life Technologies [11], and HPG-Aligner, an open access mapper designed by Joaquín Tárraga et al. [12].

## 2. Materials and Methods

In this study, 217 women were recruited to evaluate the performance of the Massively Parallel Sequencing- (MPS-) based test as a screening test. The inclusion criteria were pregnant women, 18 years old or over, with a singleton live fetus. The risk of aneuploidy was not an inclusion criterion. Two different groups of women were asked to participate. The main group was composed of pregnant women who decided to perform an invasive procedure. For those cases, fetal quantitative fluorescent PCR or karyotype results were achieved as part of regular clinical care. The second group was composed of pregnant women with the possibility of carrying a fetus affected with an X-linked disease. In these fetuses, the phenotype was determined after birth to exclude trisomies.

Informed written consent was obtained from each participant and ethical approvals were granted by the respective institutional boards of the participating institution.

The study includes 58 pathological samples (34 trisomy 21, 18 trisomy 18, and 6 trisomy 13) and 159 euploid samples. In Supplementary Table 1, the demographics and pregnancy-related information for the selected tested samples are described.

The peripheral blood samples for NIPT were obtained before any invasive procedure. All peripheral blood samples were processed under the same protocol. 10 mL of maternal whole blood was drawn into an EDTA-K2 collecting tube (Terumo Europe®, Spain) and was centrifuged within 24 hours of collection. Then the supernatant plasma was separated and recentrifuged. The supernatant plasma was aliquoted into 0.9 mL volumes in 1.5 eppendorf tubes and stored at -20°C until further processing.

After thawing the plasma samples, cell-free DNA was extracted from the plasma, using the QIAamp DSP virus kit (Qiagen, Hilden, Germany) as described previously [13].

The extracted cfDNA was used for library preparation in accordance with manufacturer's protocol modified for our type of samples. Libraries were prepared using the Ion Xpress™ Plus Fragment Library Kit and barcoded using the Ion Xpress Barcode Adapters 1-16 and 1-32 Kit (Life Technologies®, Carlsbad, California, US). The concentration of the libraries was measured using High Sensitivity DNA Reagents for 2100 Bioanalyzer (Agilent Technologies, Santa Clara, California, US).

The barcoded libraries were pooled in an 8-plex or 4-plex, clonally amplified with the Ion PI Template OT2 200 Kit v3 on the Ion One Touch™ 2 System (Life Technologies, Carlsbad, California, US), and the sphere enrichment was performed on the Ion OneTouch™ ES system (Life Technologies, Carlsbad, California, US) according to the manufacturer's protocol.

Template spheres were loaded on an Ion PI™ Chip v2, and sequencing was performed using the Ion PI™ Sequencing 200 Kit v3 on an Ion Proton™ System (Life Technologies, Carlsbad, California, US) running Torrent Suite™ Software 4.2.

The data were initially processed with the Ion Torrent platform-specific pipeline software (Torrent Suite Software 4.2) to generate sequence reads, trim adapter sequences, and filter out low-quality reads.

Ion Torrent's mapping program (TMAP, version 4.2; https://github.com/iontorrent/TMAP) was used to align the generated sequence data to the hg19, GRCh37 human reference genome obtained from the application, with the parameters –a 0 stage1 map4.

The same unaligned FASTQ were processed with the HPG-Aligner pipeline software (https://github.com/opencb/hpg-aligner) using the hg19, GRCh37 (UCSC Genome Browser), with the default parameters.

Both mappers are suitable for short and long nucleotide sequences but use different approaches. TMAP is based on the Burrows-Wheeler Transform and uses several algorithms such as BWA, BWASW, SSAHA2, and the super maximal exact matching algorithm [11], whereas HPG is based on the combination of the performance of uncompressed Suffix arrays with the Smith-Waterman algorithm [12].

Sequences that could be mapped to just one location in the reference human genome were counted per chromosome using Samtools version 0.1.19-44428cd (https://github.com/samtools/samtools.git).

Fetal DNA fraction was estimated through size analysis of maternal plasma DNA by sequencing as described by Yu* et al.* (2014) [14]. The size of each sequenced plasma DNA molecule was calculated using Biopieces. Then the size ratio indicating the relative proportions of short and long DNA fragments was calculated for each sample. This ratio correlates with the fetal DNA fraction with a positive linear relationship [14].

To ensure the pathogenicity of the samples, in each experiment, three different scores for chromosomes 13, 18, and 21 were calculated and compared against the normal range: (a) the Z score, described by Chiu et al. (2008) [15]; (b) a score based on a modification of the estimation of DNA fraction, described by Fan et al. (2008), called from now on Trisomy Ratio (TR) [16]; and (c) the Fractional Genomic Representation (FGR) described by Lau et al. (2012) [17]. The last two approaches are taking into account the chromosome content of GC and, in all the scores, samples from two euploid pregnancies were used as reference (more details in supplementary material).

In each experiment, the relative proportion of DNA fragments from a specific chromosome, 21, 13, and 18, is estimated and compared against the normal range, using a ROC curve (IBM SPSS Statistics 20), employing the values of 34 samples with trisomy 21, 18 samples with trisomy 18, 5 with trisomy 13, and 103 euploid samples.

Different combinations of these three scores were made to determine which afforded the best sensitivity and specificity for both mappers.

The false positive, false negative, and incorrectly identified samples were sequenced again in a 4-plex experiment, when there was a sufficient amount of library or sample.

## 3. Results

In the sequencing reactions, a mean of 8,990,435 reads per sample was obtained. Also, a mean read length of approximately 145 nucleotides was achieved, which is consistent with the length distribution of plasma cell-free DNA reported by Lo* et al.* [18].

The fetal DNA fraction estimated from the size ratio described by Yu* et al.* of all the samples analyzed was above the cut-off value considered necessary for the analysis (size ratio >0.84) [14].

### 3.1. Cut-Off Values

The cut-off values for each score calculated, described in material and methods, and the sensibility and specificity of each combination for both mappers are described in Supplementary Tables 2 and 3.

Considering both mappers, the best approach with less false positive and negative values was to have values greater than the cut-off of the TR and the FGR at the same time (Supplementary Table 3).

To estimate the minimal amount of unique sequencing reads necessary to correctly identify the samples, the sensibility and specificity of each mapper according to the millions of reads were calculated (Supplementary Table 4). Based on it, when the number of unique sequencing reads after alignment and filtering was set to more than 1 million, the values for specificity and sensibility for trisomies 21, 18, and 13 are comparable to the cut-off on more than 2 million and better than when the cut-off is set on more than 3 or 4 million reads.

### 3.2. Sequencing Results

In the 8-plex experiment aligning the reads with TMAP, 203 samples were correctly classified: 148 euploid samples, 33 samples of trisomy 21 (sensibility 97.06% and specificity 99.45%), 16 samples of trisomy 18 (sensibility 88.89% and specificity 93.97%), and 6 samples of trisomy 13 (sensibility 100% and specificity 100%). There were 11 false positives of trisomy 18, 1 false negative of trisomy 18, and 2 pathological samples incorrectly identified. The sensitivity of detecting a pathological sample was 98.28% and the specificity was 93.08%.

On the other hand, using HPG-Aligner, 212 of the samples were correctly identified: 154 euploid samples, 34 samples of trisomy 21 (sensibility 100% and specificity 98.91%), 18 samples of trisomy 18 (sensibility 100% and specificity 98.99%), and 6 samples of trisomy 13 (sensibility 100% and specificity 99.53%). There were 5 false positives, 2 of trisomy 21, 2 of trisomy 18, and 1 of trisomy 13. There were no samples incorrectly identified. The sensitivity of detecting a pathological sample was 100% and the specificity was 96.86%.

After resequencing the incorrectly classified samples in a 4-plex experiment to increase the number of read counts, the number of false positives with TMAP decreases from eleven to seven, the false negative disappears, and the identification error of one of two samples was corrected. With HPG-Aligner the false positives decrease from four to two (as one of them was not resequenced due to the lack of enough sample).

**Figure 1:**
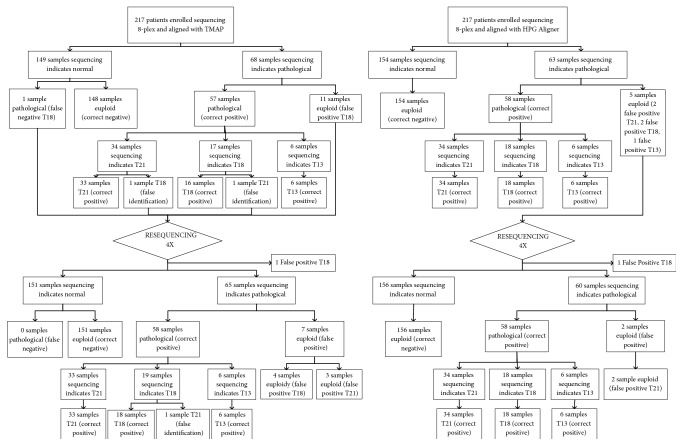
Results of NIPT for common aneuploidies using maternal plasma DNA by massively parallel Bioconductor sequencing.

The results are shown in Figure 1.

## 4. Discussion

Massively parallel DNA sequencing is becoming a part of routine clinical practice. This study was carried out by semiconductor sequencing. Although most companies use sequencing-by-synthesis platforms for aneuploidy detection in maternal plasma, recent studies demonstrate that semiconductor sequencing could be successfully used for NIPT [19–26]. This technology presents some great advantages, the most important of which is to reduce significantly the time of sequencing, a critical parameter for prenatal diagnosis. Other benefits are the lower cost per sample and its flexibility, which allows processing different number of samples depending on the chip used.

The general pipeline for analyzing NGS data has three main steps: base calling, alignment of sequence reads to a reference genome, and variant detection and genome annotation. In the pipeline of NIPT only the first two steps are followed for obtaining results. Then, after the mapped reads are counted, different statistical methods are applied for the assessment of aneuploidy.

Base calling is a process of identifying nucleotide sequences of DNA templates from different signals, such as fluorescence intensity or changes in the pH, produced by sequencers. In this step the user has to deal with the base calling algorithms that the platform provider has implemented, and in the majority of platforms the user has no access to the raw data [27].

On the other hand, there are more than 60 solutions available for the alignment of the sequence reads [10]. In the process of alignment, the most common problem arises from reads that map to multiple locations on the reference genome. Three different strategies could be used to cope with multireads. The first one is to discard all multireads and only utilize reads that map uniquely to the reference genome. This strategy, used in the majority of software programs designed for NIPT, can cause the omission of up to 30% of mappable reads [27]. The other two strategies, based on assigning the read to the location with the fewest mismatches or report all alignments until the predefined maximum number is reached, can introduce incorrect alignments and are not recommended for NIPT. Despite this, many laboratories use packaged solutions for sequencing data alignment, in which it is not well known how the software works. To study the relevance of this question, the same unaligned files were aligned with two different mappers: TMAP, provided by default from Ion Torrent Suite Software, and HPG-Aligner, an open access mapper.

In our series, TMAP aligned more than 95% of the reads obtained after sequencing (97.42%±3.46), whereas HPG only aligned 84.65%±4.72, with a difference intrasample for each mapper between 2.46 and 23% (12.76±3.05). These data suggest that HPG is more restrictive with the reads performing a better alignment of them; while TMAP could increase the number of mislocated read, with worse results as a consequence.

For the determination of the efficiency of each mapper in NIPT, different scores described in the literature were tested [15–17]. Most of the series published for NIPT based on semiconductor sequencing use only the Z-score to classify their results [19, 23, 25]. This score does not take into account the cytosine and guanine content of each chromosome. On the contrary, the TR and FGR, calculated also in this study, consider the cytosine and guanine content. As none of the three scores were accurate enough to be used alone, different combinations were compared. Having a positive score for the TR and the FGR was the best approach to detect a possible trisomy.

It is not clear how many reads per sample are required to detect aneuploidies. Not all the publications refer the cut-off for the minimal number of reads used. Dheedene et al. resequenced with less than 5M read [19], while Liao et al. required a minimum of 3.5M reads for the classification [25]. In our study the minimal amount of reads necessary, after mapping and filtering, was reduced to one million without a considerable loss of sensitivity or specificity (Supplementary Table 4). Decreasing the cut-off for the number of reads reduces the number of noninformative samples and allows cutting down the cost.

Most studies by semiconductor sequencing use the Ion Torrent's mapping program or other mappers based also on BWA algorithm [19, 23–26]. According to our results, HPG-Aligner has less false positives than TMAP. In addition, by using HPG-Aligner, there are neither false negative results nor samples incorrectly identified. All the samples incorrectly classified were collected in an appropriate gestational window (11-19 gestation weeks) and have no physiological factors that can influence these results (Supplementary Table 5).

In order to determine if the result errors were due to an insufficient amount of reads, these samples were resequenced in a 4-plex experiment to double the reads counts. After the analysis of the new resequencing results, the number of false positives using HPG-Aligner was reduced by half, whereas applying TMAP the false positive decreases from eleven to seven, the false negative disappears, and only one sample was incorrectly classified. It seems that both mappers work better with a higher coverage, but only for the 17 samples incorrectly classified, six have less than 5 million reads aligned. After resequencing them, only one sample has been well reclassified with HPG-Aligner but not with TMAP (sample 3_6), although the number of reads has increased to 16.3 Million (Supplementary Table 5). These results point out that sequencing the samples with a higher coverage does not improve the results. Based on it, a cut-off value of 1 million reads is valid to give correct results with a sensitivity for detecting aneuploidy of 98.28% and specificity of 93.08% for TMAP and 100% and 96.86% for HPG-Aligner (Supplementary Table 4). In the unique false positive value common to both mappers, the diagnostic sample was amniotic fluid, so this could be a result of a confined placental mosaicism (CPM). One study shows that the incidence of CPM could be 4.8% [8]. Regarding this, confirmation using an invasive test remains necessary, at least when the woman is considering an irreversible decision [3, 28]. According to this, and taking into account the fact that cffDNA comes from placenta, amniocentesis would be a more appropriate and reliable follow-up diagnostic test than CVS in case of positive NIPT, especially if there is an absence of sonographic features in the fetus suggestive of trisomy [29–31].

In this study, the samples were collected from general population in a real clinical setting with an appropriate gestational window. Many studies of NIPT by semiconductor sequencing are focused exclusively on high risk pregnant women [20–24], but our results point out the potential use not only in women with high risk of having a fetus with a chromosomal abnormality, but also for low or no-risk pregnancies. The advantages of semiconductor sequencing against other platforms, such as short time consumed, lower cost, and flexibility [26], make this technology affordable and scalable for noninvasive aneuploidy screening in general population.

## 5. Conclusion

In summary, this is the first study that compares two different read mappers for NIPT of common aneuploidies in general population by semiconductor sequencing. Significant and important differences between the two mappers were identified. In particular, HPG-Aligner provides less false positive results than TMAP and, more importantly, no false negative results. The correct choice of mapper is crucial in next-generation sequencing data analysis, although more studies are needed to reach a consensus as to which mapper is the best for each application and data type.

## Data Availability

Under request the data related to this article will be provided.

## Supplementary Materials

Supplementary 1 Supplementary table 1: demographics and pregnancy‐related information for the selected samples tested.

Supplementary 2 Supplementary table 2: cut‐off values from ROC curves for three different scores.

Supplementary 3 Supplementary table 3: sensitivity and specificity for each score.

Supplementary 4 Supplementary table 4: sensibility and specificity of each mapper according to the minimum millions of unique reads.

Supplementary 5 Supplementary table 5: biological, demographical, and sequencing data from the samples incorrectly classified.
