# Supplementary material for: Noninvasive Prenatal Testing: Comparison of Two Mappers and Influence in the Diagnostic Yield
Source: Biomed Res Int. 2018 Jun 7;2018:9498140. doi: 10.1155/2018/9498140 (PMC6011118; doi:10.1155/2018/9498140)
Supplement: Supplementary 1 — Supplementary table 1: demographics and pregnancy‐related information for the selected samples tested. [file 9498140.f1.pdf]

Supplementary Table 1. Demographics and pregnancy-related information for the selected samples tested.

**Characteristic**	**Euploid (N=159)**	**Aneuploid (N=58)**	**p**
**Maternal age, years (average, SD)**	35.126 ± 4.932 (N=159)	36.190 ± 4.331 (N=58)	P=0.126
<35 (N, %)	58/159 (36.5%)	21/58 (36.2%)	
≥35 (N,%)	101/159 (63.5%)	37/58 (63.8%)	
**Gestational age, weeks (average, SD)**	13.90 ± 2.30 (N=159)	12.74 ± 1.86 (N=58)	P<0.001
**Racial origin (N, %)**			P=0.379
Caucasian	137/155 (88.4%)	43/56 (76.8%)	
South American	8/155 (5.2%)	11/56 (19.6%)	
Asian	5/155 (3.2%)	0/56 (0%)	
African	5/155 (3.2%)	2/56 (3.6%)	
**Weight, kg (average, SD)**	61.85±9.72 (N=143)	64.41±12.80 (N=54)	P=0.188
**MBI, kg/m2 (average, SD)**	23.70±6.64 (N=129)	24.14±4.75 (N=52)	P=0.542
**Current smoker (N, %)**	16/144 (11.11%)	4/55 (7.2%)	P=0.385
**Maternal diabetes mellitus (N, %)**	2/144 (1.4%)	0/58 (0%)	
**Risk of T21** †			P<0.001
Without risk (<1:1000)	37/142 (26.1%)	1/53 (1.9%)	
Intermediate risk (1:1000-1:250)	18/142 (12.7%)	4/53 (7.5%)	
High risk (>1:250)	87/142 (61.2%)	48/53 (90.6%)	

**Risk of T18** †			P<0.001
Without risk (<1:1000)	113/126 (89.7%)	10/50 (20.0%)	
Intermediate risk (1:1000-1:250)	3/126 (2.4%)	2/50 (4.0%)	
High risk (>1:250)	10/126 (7.9%)	38/50 (76.0%)	
**Main indication (N, %)**			P<0.001
Positive combined screening (>1:250)	82/159 (51.6%)	30/58 (51.8%)	
Abnormal fetal ultrasound scan	7/159 (4.4%)	26/58 (44.8%)	
Positive family history	13/159 (8.2%)	0/58 (0%)	
Advanced maternal age	28/159 (17.6%)	1/58 (1.7%)	
Other or not indicated	29/159 (18.2%)	1/58 (1.7%)	
**Diagnostic procedure (N, %)**			P<0.001
Chorionic villus sampling	53/159 (33.3%)	49/58 (84.5%)	
Amniocentesis	91/159 (57.2%)	9/58 (15.5%)	
Fetal tissues	2/159 (1.3%)	0/58 (0%)	
Phenotypic features in newborn	13/159 (8.2%)	0/58 (0%)	
**Diagnostic technique**			P=0.018
QF-PCR	6/159 (3.8%)	4/58 (6.9%)	
Karyotyping	0/159 (0%)	3/58 (5.2%)	
Both	140/159 (88.0%)	51/58 (87.9%)	

Phenotypic features of euploid in the neonates	13/159 (8.2%)	0/58 (0%)	
**Fetal sex**			0.655
Male	74/159 (46.5%)	29/58 (50.0%)	
Female	85/159 (53.5%)	29/58 (50.0%)	

For some patients not all information was available. Number of patients used to calculate statistics is indicated for each parameter.

†Risk ratios that Hudecova defines in the study published in 2014 were used to determine the risk of aneuploidy.[1]

[1] Hudecova et al., «Maternal Plasma Fetal DNA Fractions in Pregnancies with Low and High Risks for Fetal Chromosomal Aneuploidies».
