# Supplementary material for: Noninvasive Prenatal Testing: Comparison of Two Mappers and Influence in the Diagnostic Yield
Source: Biomed Res Int. 2018 Jun 7;2018:9498140. doi: 10.1155/2018/9498140 (PMC6011118; doi:10.1155/2018/9498140)
Supplement: Supplementary 2 — Supplementary table 2: cut‐off values from ROC curves for three different scores. [file 9498140.f2.pdf]

Supplementary table 2. Cut-off values for each score obtained by ROC curves.

Z-Score

		Z-SCORE	
		**TMAP**	**HPG**
TRISOMY 21		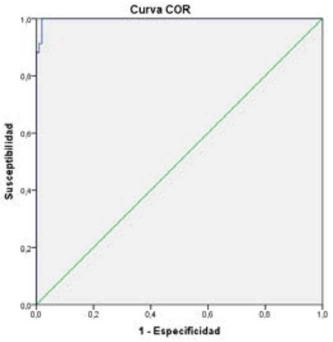	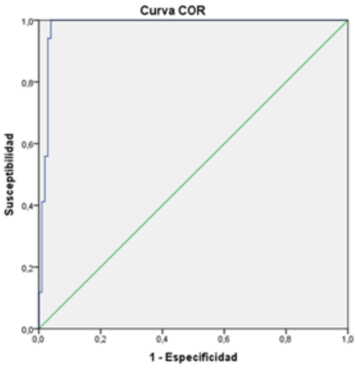
	AUC	0.998	0.981
	CUT-OFF VALUE	1.5456	1.9532
	SENSITIVITY	100%	100%
	SPECIFICITY	98.00%	96.10%
TRISOMY 18		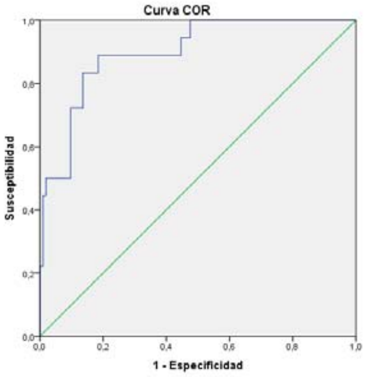	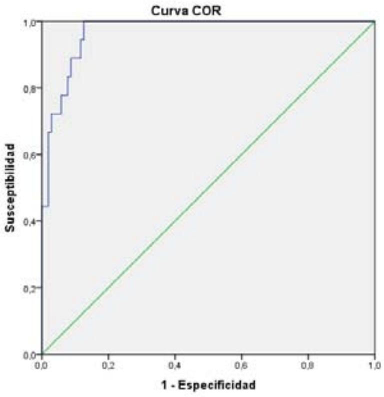
	AUC	0.889	0.968
	CUT-OFF VALUE	1.0117	1.1774
	SENSITIVITY	83.30%	100%

	SPECIFICITY	86.40%	87.40%
TRISOMY 13		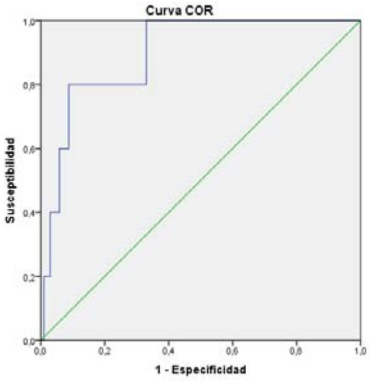	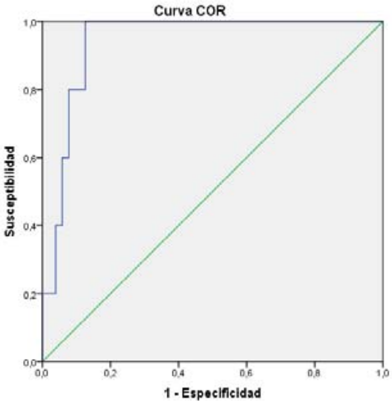
	AUC	0.897	0.94
	CUT-OFF VALUE	1.1564	0.8283
	SENSITIVITY	80%	100%
	SPECIFICITY	91.30%	87.40%

Trisomy ratio

		**TR**	
		**TMAP**	**HPG**
TRISOMY 21		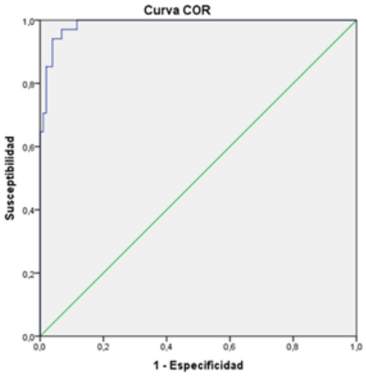	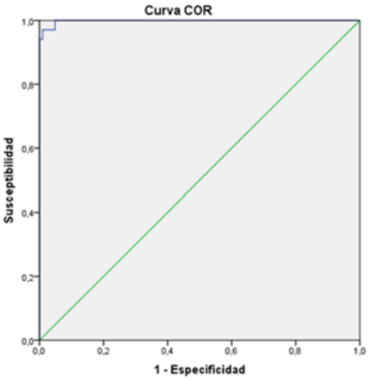
	AUC	0.988	0.998

	CUT-OFF VALUE	100.9884	101.2099
	SENSITIVITY	100%	100%
	SPECIFICITY	88.40%	95.10%
TRISOMY 18		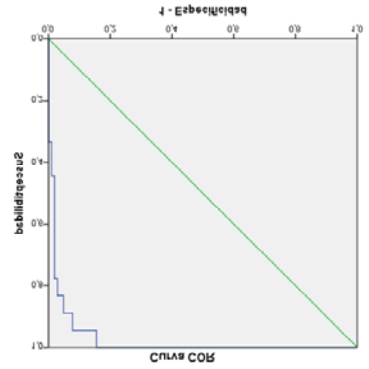	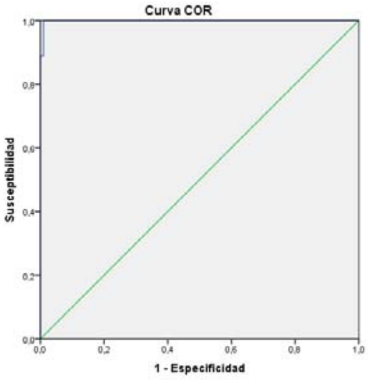
	AUC	0.975	0.999
	CUT-OFF VALUE	101.3703	101.4261
	SENSITIVITY	100%	100%
	SPECIFICITY	84.50%	99.03%
TRISOMY 13		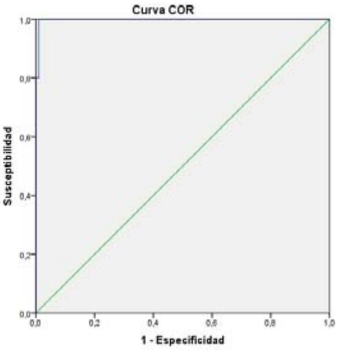	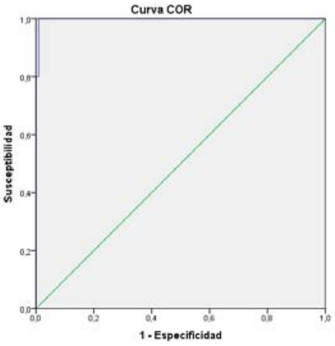
	AUC	0.998	0.998
	CUT-OFF VALUE	101.2612	101.4996
	SENSITIVITY	100%	100%
	SPECIFICITY	99%	99%

Fractional Genomic Representation

		FGR	
		**TMAP**	**HPG**
TRISOMY 21		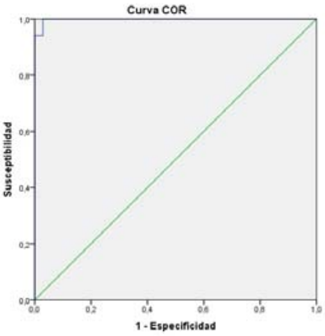	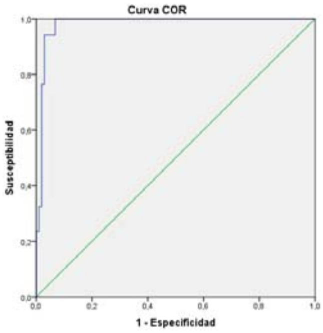
	AUC	0.998	0.981
	CUT-OFF VALUE	1.8782	2.1542
	SENSITIVITY	100%	100%
	SPECIFICITY	97.10%	93.20%
TRISOMY 18		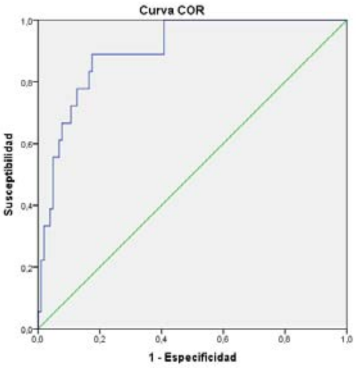	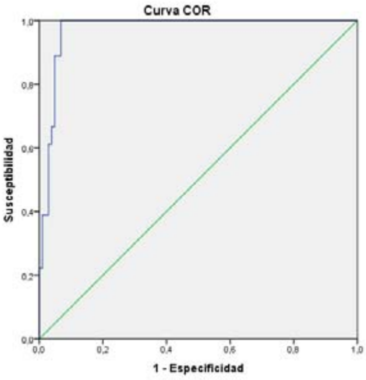
	AUC	0.901	0.971
	CUT-OFF VALUE	0.9522	3.04
	SENSITIVITY	88.90%	100%

	SPECIFICITY	82.50%	94.20%
TRISOMY 13		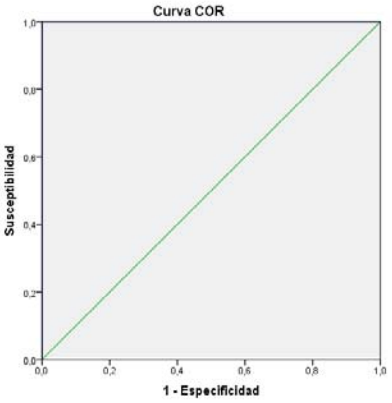	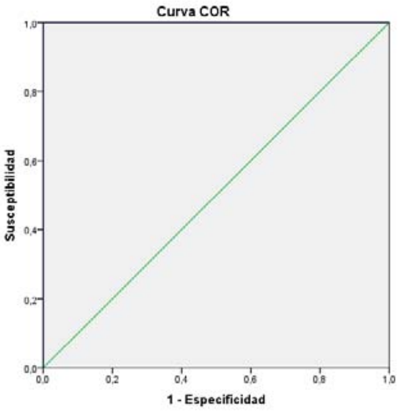
	AUC	1	1
	CUT-OFF VALUE	6.7248	5.1534
	SENSITIVITY	100%	100%
	SPECIFICITY	99%	100%
