# Supplementary material for: Noninvasive Prenatal Testing: Comparison of Two Mappers and Influence in the Diagnostic Yield
Source: Biomed Res Int. 2018 Jun 7;2018:9498140. doi: 10.1155/2018/9498140 (PMC6011118; doi:10.1155/2018/9498140)
Supplement: Supplementary 3 — Supplementary table 3: sensitivity and specificity for each score. [file 9498140.f3.pdf]

Supplementary Table 3. Sensitivity and specificity for each ratio.

	**Z-SCORE**		**TR**		**FGR**		**TR AND Z-SCORE OR FGR**		**TR AND FGR**		**TR AND Z-SCORE**		**Z-SCORE AND FGR**		**THREE SCORES**	
	**TMAP**	**HPG**	**TMAP**	**HPG**	**TMAP**	**HPG**	**TMAP**	**HPG**	**TMAP**	**HPG**	**TMAP**	**HPG**	**TMAP**	**HPG**	**TMAP**	**HPG**
SENSITIVITY FOR TRISOMY 21 (%)	91.2	91.2	94.1	100.0	91.2	100.0	97.1	100.0	97.1	100.0	97.1	97.1	97.1	100.0	97.1	100.0
SPECIFICITY FOR TRISOMY 21 (%)	94.0	92.9	92.9	96.2	95.6	94.5	99.5	96.7	99.5	98.9	99.5	98.4	96.7	98.9	99.5	99.5
SENSITIVITY FOR TRISOMY 18 (%)	61.1	94.4	100.0	100.0	61.1	83.3	88.9	100.0	88.9	100.0	88.9	100.0	72.2	100.0	83.3	100.0
SPECIFICITY FOR TRISOMY 18 (%)	92.5	94.0	89.4	97.5	87.9	98.5	94.0	99.0	94.0	99.0	95.0	98.0	92.5	99.0	94.5	99.0
SENSITIVITY FOR TRISOMY 13 (%)	50.0	100.0	100.0	100.0	100.0	83.3	100.0	100.0	100.0	100.0	83.3	100.0	83.3	100.0	83.3	100.0
SPECIFICITY FOR TRISOMY 13 (%)	91.9	91.0	98.6	99.1	97.2	97.6	100.0	99.1	100.0	99.5	100.5	99.1	99.1	99.1	100.0	99.5
SENSITIVITY FOR ANEUPLOIDY (%)	93.1	100.0	98.3	100.0	96.6	100.0	98.3	100.0	98.3	100.0	94.8	98.3	94.8	100.0	94.8	100.0
SPECIFICITY FOR ANEUPLOIDY (%)	78.6	74.8	77.4	91.2	81.1	91.2	93.1	93.7	93.1	96.9	94.3	94.3	88.1	96.2	93.7	97.5
FALSE POSITIVES	35	40	37	14	31	14	11	10	11	5	10	10	19	6	10	4
FALSE NEGATIVES	3	0	0	0	1	0	1	0	1	0	2	0	3	0	3	0
