# Supplementary material for: Noninvasive Prenatal Testing: Comparison of Two Mappers and Influence in the Diagnostic Yield
Source: Biomed Res Int. 2018 Jun 7;2018:9498140. doi: 10.1155/2018/9498140 (PMC6011118; doi:10.1155/2018/9498140)
Supplement: Supplementary 4 — Supplementary table 4: sensibility and specificity of each mapper according to the minimum millions of unique reads. [file 9498140.f4.pdf]

Supplementary Table 4. Sensibility and specificity of the mappers according to the minimum millions of unique reads.

	All the samples regardless of the number of reads		>1 Million reads		>2 Million reads		>3 Million reads		>4 Million reads	
	**TMAP**	**HPG Aligner**	**TMAP**	**HPG Aligner**	**TMAP**	**HPG Aligner**	**TMAP**	**HPG Aligner**	**TMAP**	**HPG Aligner**
**Sensitivity Trisomy 21**	97.06	100.00	97.06	100.00	97.06	100.00	96.88	100.00	96.77	100.00
**Specificity Trisomy 21**	99.46	98.39	99.45	98.91	99.44	98.88	99.42	98.84	99.39	98.79
**Sensitivity Trisomy 18**	88.89	100.00	88.89	100.00	88.89	100.00	88.24	100.00	88.24	100.00
**Specificity Trisomy 18**	94.06	99.01	93.97	98.99	94.33	98.97	94.12	98.93	93.85	98.88
**Sensitivity Trisomy 13**	100.00	100.00	100.00	100.00	100.00	100.00	100.00	100.00	100.00	100.00
**Specificity Trisomy 13**	100.00	99.53	100.00	99.53	100.00	99.51	100.00	99.49	100.00	99.47
**Sensitivity aneuploidy**	98.28	100.00	98.28	100.00	98.28	100.00	98.18	100.00	98.15	100.00
**Specificity aneuploidy**	93.21	96.30	93.08	96.86	93.51	96.75	93.29	96.64	92.96	96.48
