# Supplementary material for: Noninvasive Prenatal Testing: Comparison of Two Mappers and Influence in the Diagnostic Yield
Source: Biomed Res Int. 2018 Jun 7;2018:9498140. doi: 10.1155/2018/9498140 (PMC6011118; doi:10.1155/2018/9498140)
Supplement: Supplementary 5 — Supplementary table 5: biological, demographical, and sequencing data from the samples incorrectly classified. [file 9498140.f5.pdf]

Sample	Reference sample	QF-PCR/ karyotipe	[pmol/l]	Secuencing results							
				Reads	%chip	NRA TMAP	% reads mapped TMAP	NRA HPG	% reads mapped HPG	TR+FGR TMAP	TR+FGR HPG
10_5	CVS	47,XX+18	193.8	7,021,412	9.16	6,672,572	95.03	5,937,822	84.57	Incorrectly identified T21	Trisomy 18
15_3	AF	46,XY	27.1	5,538,611	6.50	5,286,437	95.45	4,670,419	84.32	False Positive T18	Euploid
18_33	AF	46,XY	7.97	2,774,019	9.52	2,595,204	92.47	2,084,577	75.15	Euploid	False Positive T21
19_4	AF	46,XX	32.4	5,596,764	7.49	5,417,211	96.79	4,778,337	85.38	False Positive T18	Euploid
20_22	AF	46,XY	50.8	11,323,073	14.51	11,020,461	97.33	9,531,601	84.18	False Positive T18	False Positive T21
21_31	CVS	46,XX	10.87	15,562,358	20.94	15,231,058	97.87	13,212,672	84.90	False Positive T18	Euploid
22_20	AF	46,XY	5.59	9,509,391	11.95	9,186,265	96.60	8,192,557	86.15	Euploid	False Positive T18
25_25	AF	46,XY	8.47	11,788,291	14.85	11,436,216	97.01	10,151,560	86.12	Euploid	False Positive T13
26_30	AF	46,XY	28.8	1,089,537	1.43	1,050,484	96.42	923,187	84.73	False Positive T18	Euploid
3_4	PH	Euploid	651.9	6,457,932	7.63	6,077,578	94.11	5,119,264	79.27	False Positive T18	Euploid
3_6	AF	46, XY	6061.1	4,478,886	5.27	4,202,795	93.84	3,579,404	79.92	False Positive T18	False Positive T18
3_7	AF	46, XY	208.7	8,242,646	9.67	7,709,927	93.54	6,570,885	79.72	False Positive T18	Euploid
3_9	CVS	47,XY+21	7477.7	3,307,346	4.60	3,032,347	91.69	2,491,380	75.33	Incorrectly identified T18	Trisomy 21
4_2	CVS	46,XY	297.2	4,809,940	8.43	4,595,986	95.55	3,899,508	81.07	False Positive T18	Euploid
6_7	CVS	46,XY	594.6	2,131,689	2.80	2,066,603	96.95	1,821,440	85.45	False Positive T18	Euploid
7_5	AF	46,XY	1209	7,322,010	9.44	6,982,254	95.36	6,243,977	85.28	False Positive T18	Euploid
9_11	CVS	47,XY+18	432.1	8,557,813	9.31	8,125,563	94.95	7,244,362	84.65	False Negative T18	Trisomy 18

CVS: Chorionic villus sampling
AF: amniotic fluid
PH: phenotiphic features in new born
NRA: number of reads aligned
[pmol/l]: library concentration

Sample	Resequencing reults							
	Reads	%chip	NRA TMAP	% reads mapped TMAP	NRA HPG	% reads mapped HPG	TR+FGR TMAP	TR+FGR HPG
10_5	20,423,254	26.79	19,897,179	97.42	17,333,555	84.87	Trisomy 18	Trisomy 18
15_3	13,487,649	17.66	13,118,185	97.26	11,478,468	85.10	False Positive T21	Euploid
18_33	8,318,533	10.85	8,111,860	97.52	7,015,960	84.34	Euploid	False Positive T21
19_4	14,479,851	18.99	14,055,006	97.07	12,243,590	84.56	False Positive T18	Euploid
20_22	18,788,320	24.75	18,225,235	97.00	15,803,561	84.11	False Positive T21	False Positive T21
21_31	15,983,445	21.10	15,598,679	97.59	13,569,724	84.90	Euploid	Euploid
22_20	-	-	-	-	-	-	-	-
25_25	15,246,382	19.88	14,845,806	97.37	13,092,597	85.87	Euploid	Euploid
26_30	-	-	-	-	-	-	-	-
3_4	12,302,992	14.70	11,714,013	95.21	10,189,946	82.82	Euploid	Euploid
3_6	17,201,588	28.39	16,284,267	94.67	14,483,653	84.20	False Positive T18	Euploid
3_7	12,352,404	15.58	11,757,551	95.18	10,463,484	84.71	Euploid	Euploid
3_9	4,304,845	5.09	4,056,035	94.22	3,432,628	79.74	Incorrectly identified T18	Trisomy 21
4_2	15,376,969	19.37	14,611,825	95.02	13,142,061	85.47	False Positive T21	Euploid
6_7	6,557,563	8.74	6,355,032	96.91	5,599,505	85.39	False Positive T18	Euploid
7_5	19,365,845	13.18	18,478,540	95.42	15,908,609	82.15	False Positive T18	Euploid
9_11	15,573,621	25.70	14,737,517	94.63	13,078,644	83.98	Trisomy 18	Trisomy 18

Sample	Main indication	Risk of T21	Risk of T18	Maternal age	MBI (kg/m2)	Weigth (Kg)	Current smoker	Maternal diabetes mellitus	Racial origin	Gestational age (weeks)
10_5	Abnormal ultrasound scan	1:50	1:50	34	19.6	52	No	No	Caucasian	11
15_3	Positive combined screening	1:23	<1:10000	34	21.6	52	No	No	Caucasian	12
18_33	Advanced maternal age	-	-	40	34.4	88	No	Sí	Caucasian	15
19_4	Positive combined screening	1:161	<1:10000	36	30.0	72	No	No	Caucasian	17
20_22	Other	1:317	<1:10000	40	24.6	54	No	No	Caucasian	18
21_31	Positive combined screening	1:16	<1:10000	35	17.8	55	No	No	Caucasian	13
22_20	Other: maternal anxiety	1:3499	<1:10000	38	20.5	48	No	No	Caucasian	15
25_25	Positive combined screening	1:150	<1:10000	38	24.8	65	No	No	Caucasian	15
26_30	Positive family history	1:868	<1:10000	34	24.0	60	No	No	South America	14
3_4	Anti-D isoimmunization	<1:10000	<1:10000	29	26.3	64	No	No	South America	19
3_6	Advanced maternal age	1:1582	<1:10000	41	22.5	57	No	No	Caucasian	15
3_7	Positive combined screening	1:618	1:62	36	26.1	66	No	No	Caucasian	16
3_9	Positive combined screening	1:45	1:41	39	32.0	73	No	No	Caucasian	12
4_2	Positive combined screening	1:116	<1:10000	26	27.1	66	No	No	Caucasian	12
6_7	Positive combined screening	1:5	<1:10000	42	26.1	60	No	No	Caucasian	13
7_5	Positive family history	-	-	30	23.4	60	No	No	-	16
9_11	Abnormal ultrasound scan	1:6	1:3	40	28.1	72	No	No	Caucasian	12
